# Birth Preparedness and Complication Readiness Practice and Associated Factors among Pregnant Women, Northwest Ethiopia

**DOI:** 10.1155/2016/8727365

**Published:** 2016-09-18

**Authors:** Yewondwossen Bitew, Worku Awoke, Simachew Chekol

**Affiliations:** ^1^GAMBY College of Medical Sciences, P.O. Box 209, Bahir Dar, Ethiopia; ^2^College of Medicine and Health Sciences, Bahir Dar University, P.O. Box 693, Bahir Dar, Ethiopia

## Abstract

*Background*. Little is known about birth preparedness and complication readiness (BPCR) plan in resource limited settings to decrease maternal mortality. Therefore, this study was done to assess the status of BPCR and associated factors among pregnant women in South Wollo, Northwest Ethiopia, by involving 819 pregnant women from March to April, 2014. Data were collected by using pretested interviewer administered questionnaire and analyzed using a computer program of SPSS version 20.00.* Results*. Pregnant women who were prepared for at least three elements of BPCR were 24.1%. Pregnant women knowing at least three key danger signs during pregnancy, delivery, and postnatal period were 23.2%, 22.6%, and 9.6%, respectively. Women having secondary education and higher were 6.20 (95% CI = [1.36, 28.120]) times more likely to be prepared than illiterates. Women having a lifetime history of stillbirth [5.80 (1.13, 29.63)], attending ANC for last child pregnancy [5.44 (2.07, 14.27)], participating in community BPCR group discussion [4.36 (1.17, 16.26)], and having their male partner involved in BPCR counseling during ANC follow-up [4.45 (1.95, 10.16)] were more likely to be prepared.* Conclusions*. BPCR was very low and should be strengthened through health communication by involving partner in BPCR counseling.

## 1. Background

Every pregnancy faces risks and every minute of every day, somewhere in the world, a woman dies as a result of complications arising during pregnancy and childbirth. The majority of these deaths are avoidable by accessing quality maternal health service [1, 2]. Globally, in year 2010, an estimated 287 000 maternal deaths occurred. Sub-Saharan Africa and Southern Asia accounted for 85% of the global burden (245,000 maternal deaths). Maternal mortality in developing regions was 15 times higher than in developed regions. Most of them died because they had no access to skilled routine and emergency care [3].

Ethiopia as a sub-Saharan African country is characterized by very high maternal mortality, 676 maternal deaths per 100,000 live births, low coverage of ANC (34%), and low coverage of skilled delivery and immediate postpartum care (10% and 7%, resp.) [4]. About fifteen percent of pregnant women in Ethiopia are estimated to develop obstetric complications which are potentially life-threatening. An estimated 2.6 million births occur each year. Direct complication accounts for 85% of the deaths as well as many acute and chronic illnesses [5]. Maternal deaths in Ethiopia account for 30 percent of all deaths in women age 15–49 [4].

Reduction of maternal mortality is a global priority and it is one of the millennium development goals [6]. The key to reducing maternal mortality ratio and improving maternal health is increasing attendance by skilled health personnel throughout pregnancy and delivery [7]. Birth preparedness and complication readiness is one of keys for safe motherhood strategy whose objective is to promote the timely use of skilled maternal and neonatal care during childbirth by making a birth plan and promoting active preparation and decision making for delivery of pregnant women and their families [8–11].

In Ethiopia little has been documented on BPCR practice and most studies done on safe motherhood focus on service uptake, quality, and utilization. Therefore, this paper is designed to evaluate BPCR practice and associated factors among pregnant women with second and third trimester in South Wollo of Northwest Ethiopia. This study will contribute to provide evidence based knowledge on the current status of BPCR among pregnant women and provide basic data for service providers, policy makers, development partners, and programmers to design effective BPCR program intervention in an attempt to reduce highly maternal and neonatal mortality rate in Ethiopia.

## 2. Methods

### 2.1. Ethical Considerations

Ethical clearance was obtained from Bahir Dar University and GAMBY College of Medical Sciences. A formal letter for permission and support was taken from the Amhara Regional Health Bureau to South Wollo Zone Health Department and District Health Offices. Pregnant women were informed about the objective of the study and verbal informed consent was obtained before conducting the interview. Client's privacy and confidentiality of the information was assured during the interview process. After data collected from each client, counseling and education on BPCR and key danger signs are provided for women and their families. Mothers who did not attend or discontinued the ANC services were referred to the nearby health center/hospital using a referral tool for ANC service.

### 2.2. Study Design and Period

A community-based cross-sectional study was conducted among pregnant women of second and third trimester of pregnancy in South Wollo Zone, Northwest Ethiopia, from March 1 to April 30, 2014. South Wollo Zone is one of the ten zones of Amhara National Regional State located 480 KM away from the Regional Capital, Bahir Dar.

### 2.3. Sample Size Determination

The sample size was determined using single population proportion formula. The following assumptions were considered: a level of confidence 95%, a 5% margin of error, and 50% proportion of pregnant mothers prepared for birth. Additional 10% allowance for none response rate and correction for multistage sampling design effect 2 were considered. The final sample size becomes 845 pregnant mothers. Then, the calculated sample size was proportionally allocated to districts of urban and rural Kebele (the smallest administrative unit in Ethiopia) based on the expected number of pregnant women. Each Kebele was selected using simple random sampling method and finally pregnant women were selected by systematic sampling using the updated list of pregnant women from each selected Kebele.

### 2.4. Inclusion Criteria

Those women with at least 3 months of current pregnancy, permanent resident of the study area, and who gave oral consent to participate were interviewed.

### 2.5. Exclusion Criteria

Women who were severely ill and incapable of being interviewed and those who were not able to give the oral consents to participate in the study were excluded.

### 2.6. Data Collection and Analysis

Structured questionnaires were adapted from a survey tool developed by JHPIEGO Maternal, Neonatal Health Program and translated into local Amharic language. Training was given for data collectors and supervisors on how to maintain a face-to-face interview, keeping the quality of data and ethical issues. The collected data were checked, cleaned, coded, entered, and analyzed using a computer program of SPSS version 20.00. Bivariate and multivariate logistic regression analyses were done. Crude and adjusted odds ratio (OR) with a corresponding 95% CI (confidence interval) was calculated to determine relationships between predictor variables and practice of birth preparedness and complication readiness.

## 3. Results

### 3.1. Sociodemographic Characteristics

A total number of 845 pregnant women of second and third trimester were approached and 819 participated making the response rate 96.9%. As shown in Table 1, 84.1% of women were from the rural area, 93.5% of them were married by their marital status, 76.4% were Muslim by their religion category, and 81.1% were housewives. The majority (60.2%) has an average monthly income of less than 1000 Ethiopian birr (Table 1).

### 3.2. Obstetric Characteristics of Respondents

The majority of (77.2% and 86.9%) the mothers were gravida and parity of five and above, respectively. Before the current pregnancy, only 39.6% of the respondents received ANC at health center and hospital level and 60.4% did not get ANC for last child pregnancy. Only 15.7% of women delivered at health facility and 5.9% of them experienced a lifetime history of stillbirth and only 10.1% received PNC services for last childbirth (Table 2).

### 3.3. Pregnant Women Attending Antenatal Care (ANC)

Majority (71.8%) of pregnant mothers attended the ANC service at health facility level; however only 68.0% received information about BPCR. More than half of them (66.7%) decided to give birth at the health facility and more than a quarter (26.1%) still undecided the place where they will give birth for the current pregnancy. From all mothers who attended the ANC, only 41.3% of their partners were counseled to BPCR by health care providers. According to pregnant women response, only 44.5% of their partners decided on place of birth and the majority of them decided health facility as the place of delivery (Table 3).

### 3.4. Knowledge of Respondents on Obstetric Danger Sign

According to the findings of this study, the proportions of pregnant women who know at least three danger signs during pregnancy, delivery, and postnatal period were 23.2%, 22.6%, and 9.6%, respectively (Table 4). Regarding knowledge of key danger signs, severe vaginal bleeding has been the most frequently mentioned complication by women during the following phases: pregnancy (37.4%), labor and delivery (44.6%), and postpartum period (32.1%). Prolonged labor, which is one of the top five major causes of maternal mortality and top most causes of morbidity in low-income countries, was reported by 49.7% of mothers (Table 5).

### 3.5. Birth Preparedness and Complication Readiness

The prevalence of birth preparedness and complication readiness among pregnant women prepared for birth by practicing at least three of the following variables: identify place of delivery, save money, identify skilled provider, be aware of key danger signs and act on immediately, designate decision maker, identify source of support in case of emergency, arrange transport, EMOC, blood donor, and emergency, was found to be 197 (24.1%). 366 (44.6%) reported that they identified place of delivery, 354 (43.2%) saved money, and 286 (34.9%) prepared essential items for clean and safe delivery for birth preparation. The same as that of the practice 43.7%, 37.5%, 36.6%, and 30.4% pregnant women mentioned place of delivery, saving money, preparing essential items to clean and safe delivery, and emergency fund, respectively. But arranging transport, emergency obstetric care (EMOC), and blood donor are less mentioned and practiced by mothers (Table 6).

### 3.6. Source of Information

The majority (72.2%) heard about the term BPCR and their source of information is health care providers (82.9%). More than half (60.8%) of mothers participated in community meetings and/or pregnant women group discussions BPCR health communication organized by health extension workers and health care providers working at the Primary Health Care Unit (Table 7).

### 3.7. Factors Associated with BPCR Practice

On the bivariate logistic regression analysis, variables like place of residence, educational status, occupation, travel time to reach nearby HC/hospital, income, parity, gravidity, previous lifetime history of stillbirth, history of ANC follow-up for last child pregnancy, history of institutional delivery for last childbirth, history of postnatal care for last delivery, gestational age, knowledge of pregnant women of at least three key danger signs of pregnancy and labor and delivery and postnatal period, women's participation in BPCR related community meetings and/or pregnant women's group discussions, and ANC follow-up were found to be significantly associated factors with BPCR practice.

After analysis of confounding factors educational status of secondary education and above, previous history of lifetime stillbirth, history of ANC follow-up for last childbirth, the third trimester, women's participation in BPCR community meetings and/or pregnant women's group discussion, and current ANC follow-up of two and above visits, and partner involvement in BPCR counseling during women's ANC follow-up were found to be highly significant with BPCR practice. Pregnant mothers with educational status of secondary education and above were more likely to be prepared for birth and complication by 6 times (AOR = 6.20, 95% CI = [1.36, 28.12]) as compared to pregnant women who are unable to read and write. Women who face a lifetime history of stillbirth were about 6 times (AOR = 5.80, 95% CI = [1.13, 29.63]) more likely to be prepared than women's who did not. Mothers with a history of ANC visits during the last child pregnancy were more likely to be prepared for birth and complication by 5 times (AOR = 5.44, 95% CI = [2.07, 14.27]) than women who did not undertake ANC follow-up. Women with gestational period of the third trimester were more likely prepared for birth and complication by 8 times (AOR = 7.60, 95% CI = [2.82, 20.45]). Pregnant women who were participating in community level BPCR plan meeting and/or pregnant women group discussion on BPCR plan and antenatal care follow-up were also found to be strong predictors for BPCR practice. Pregnant women who participate in community level meeting and/or pregnant women group discussion on BPCR plan were prepared for birth and complication by 4 times (AOR = 4.36, 95% CI = [1.17, 16.21]) as compared to nonparticipants. Mothers attending two-three ANC visits and four and above visits for current pregnancy were more likely prepared for BPCR practice by 6 times (AOR = 6.20, 95% CI = [2.62,14.68]) and 7.6 times (AOR = 7.69, 95% CI = [19.02,31.09]), respectively, as compared to non-ANC attendant mothers and mothers attending only one ANC visit. Those pregnant women whose partners are involved in BPCR counseling with pregnant women during ANC follow-up were prepared for BPCR practice by 4 times as compared to noncounseled married couples on women's ANC follow-up (AOR = 4.45, 95% CI = [1.95,10.16]) (Table 8).

## 4. Discussion

The study revealed that only 24.1% of currently pregnant women of second and third trimester were prepared for delivery and obstetric emergency by practicing at least three elements of BPCR (identify place of delivery, save money, prepare essential items for clean and safe delivery, identify skilled provider, be aware of key danger signs and act on immediately, designate decision maker, identify source of support in case of emergency, arrange transport, EMOC, blood donor, and emergency fund). This result is encouraging as compared to the study done at Aleta Wondo (7%) [12] and Adegerat (22%) [13] but much less as compared to Uganda (35%) [14]. Some of them had made decisions on critical components of birth preparedness and complication readiness, including identifying the place of delivery (44.6%), saving money (43.2%), preparing essential items for clean and safe delivery (34.9%), and arranging for emergency fund (30.8%). Only a few (13.3%) of pregnant women made adequate arrangements for transportation to a health facility in case of an obstetric emergency. This is far reaching as compared to Uganda, Burkina Faso, and Nigeria [14–16].

The level of BPCR preparation was significantly associated with ANC follow-up for the current pregnancy, maternal education, community level BPCR meeting and/or pregnant women's group discussion participation, previous history on ANC follow-up for last child pregnancy and lifetime history of stillbirth, gestational age, and male partner involvement in BPCR counseling on women ANC follow-up period after adjusting possible confounding factors. Regarding maternal education, secondary and above educated mothers were more likely prepared for birth and complication readiness practice by six (6) times as compared to pregnant women who are unable to read and write. The finding was consistent with studies done in Nigeria [17], Kenya [18], and Ethiopia [13]. A woman who faced a lifetime history of stillbirth was about 6 times more likely than women's who did not. This finding was in agreement with study done at Adegerat, Ethiopia [13].

Mothers, attending two-three ANC visits, were more likely prepared for BPCR practice by 6 times and 7.6 times, respectively, as compared to non-ANC attendant mothers and mothers attending only one ANC visit. Studies conducted in Aleta Wondo, Ethiopia [13], and Burkina Faso [15] showed that women who follow an optimum number of ANC visits prepared for birth and complication as compared to non-ANC. Moreover, mothers with a history of ANC practice for last child pregnancy are more likely prepared for birth and complication by 5 times than women who did not undertake ANC follow-up for last child pregnancy. This may be due to BPCR readiness counseling that women received for last child pregnancy on their ANC follow-up.

Pregnant women who participated in community level meeting and/or pregnant women group discussion on BPCR plan were prepared for birth and complication by 4 times as compared to nonparticipants. This finding revealed the effectiveness of pregnant women small group discussion and/or community level behavioral change intervention Federal Ministry of Health of Ethiopia FMOH strategy to improve service uptake of institutional delivery [19]. Pregnant women with male partner involved in BPCR counseling during ANC follow-up were prepared for BPCR practice by 4 times as compared to noncounseled married couples on women's ANC follow-up. This may show how the decision making power in the family affects BPCR practice.

Knowledge of danger signs of obstetric complication is the first step motivating women to seek timely, skilled birth attendance care at appropriate health facility. Every woman should be made aware of the likelihood of complications during pregnancy, childbirth/labor, and the postpartum periods. Women and their spouses and community members should be given all the information on danger signs [20]. The proportion of women with knowledge of at least three key danger signs each of pregnancy (23.2%), labor (22.6%), and postpartum period (9.6%) is low but encouraging as compared to Kenya (pregnancy danger sign, 6.9%) [18] and Adegerat, Ethiopia (knowledge of at least three danger signs of pregnancy (3.9%), labor and childbirth (0.4%), and postpartum period (0.4%)) [13].

Regular attendance to antenatal care in the course of pregnancy is important in monitoring the physical status of the women and the fetus, detecting diseases and complications, and providing appropriate treatment and care. It also provides an opportunity to inform and educate pregnant women about pregnancy, childbirth, and care of the newborn and therefore enable pregnant women acquire information on danger signs of pregnancy or childbirth. Furthermore, it gives an opportunity for the woman to be counseled and make an appropriate plan for delivery [10]. In the study area, majority of the women (72%) attended antenatal care from nearby health centers and hospitals. This finding is much higher and encouraging than the EDHS 2010 (34%) [4]. A large proportion, 66.7%, of women reported that they intended to give birth at the health institution, and only 7.2% planned to deliver at home and the remaining 26.6% are still undecided on the place where they plan to give birth. This finding is much encouraging as compared to the study conducted at Aleta Wondo District of Southern Ethiopia, where only 8% of pregnant women plan to deliver at health institution [12]. Though birth preparedness and complication readiness (BPCR) plan is known as a fundamental component of all antenatal care (ANC) programs to decrease maternal mortality, it is very low in such similar resource limited settings. Using such findings is vital for evidence based decision making.

## 5. Conclusion

BPCR of pregnant women in the study area was low. Pregnant women of secondary and above educational level face lifetime history of stillbirth, with previous history of ANC follow-up for last child pregnancy, gestational period of third trimester, ANC follow-up of two and above visits, participated community level BPCR discussions, and their male partner counsel with her on BPCR which were main factors associated with BPCR practice. As recommendation, the health sectors should strengthen efforts of initiating all pregnant women in getting quality and optimum numbers of ANC visits and involving their partner in BPCR counseling during women's ANC follow-up period and strengthen community level BPCR health communication with pregnant women group discussion, families, and communities and strengthen efforts of taking appropriate decision in case of complications to reach health facilities for normal or complicated childbirth.

## Figures and Tables

**Table 1:** Sociodemographic characteristics of mothers (*N* = 819) in South Wollo Zone, Northeast Ethiopia, April, 2014.

Characteristics	*N*	%
District		
Tehuledere	172	21
Kalu	257	31.4
Woreyilu	161	19.7
Borena	229	28

Residence		
Rural	689	84.1
Urban	130	15.9

Age		
15–24	306	37.4
25–34	406	49.6
≥35	107	13.1

Marital status		
Married	766	93.5
Other	53	6.5

Maternal education		
Unable to read and write	442	54
Primary education	235	28.7
Secondary education and above	142	17.3

Occupation		
Housewife	664	81.1
Employed	89	10.9
Farmer	66	8.1

Religion		
Muslim	626	76.4
Orthodox	193	23.6

Income		
≤1000 birr	493	60.2
≥1001 birr	326	39.8

Travel time		
≤one hour	464	56.7
>one hour	355	43.3

Family size		
1–3	345	42.1
4–6	326	39.8
≥7	148	18.1

**Table 2:** Obstetric characteristics of respondents in South Wollo Zone, Northeast Ethiopia, April, 2014.

Variable	*N*	%
Gravidity (*n* = 819)		
<5	632	77.2
≥5	187	22.8

Parity (*n* = 819)		
<5	712	86.9
≥5	107	13.1

History of stillbirth (*n* = 593)		
Yes	35	5.9
No	558	94.1

ANC for last child pregnancy (*n* = 593)		
Yes	235	39.6
No	358	60.4

Delivery for last child (*n* = 593)		
Home/TBA home	501	84.3
Health facility	92	15.5

PNC for last childbirth (*n* = 593)		
Yes	60	10.1
No	533	89.9

Trimester (*n* = 891)		
Second	372	45.4
Third	447	54.6

**Table 3:** Proportion of pregnant women attending antenatal care in South Wollo Zone, Northeast Ethiopia, April, 2014.

Variable	*N*	%
ANC practice for current pregnancy (*n* = 819)		
None attended	231	28.2
Attended 1st visit	318	54.1
Attended 2nd-3rd visit	164	27.8
Attended 4th and above visit	106	18.1

Receive BPCR counseling during ANC visit (*n* = 588)		
Yes	400	68
No/I do not remember	188	32

Partners counseled about BPCR (*n* = 547)		
Yes	226	41.3
No	321	58.7

Married couples' male partner decision on place of birth for current childbirth (*n* = 766)		
Yes	341	44.5
No/I do not know	425	55.5

Place of male partner decision on place of birth (*n* = 341)		
Health institution	313	91.8
Home/TBA home	28	8.2

Pregnant women's decision on place of delivery (*n* = 819)		
Health institution	546	66.7
Undecided	214	26.1
Home/TBA home	59	7.2

**Table 4:** Proportion of women (*n* = 819) reporting key danger signs of pregnancy, childbirth, and postnatal period in South Wollo Zone, Northeast Ethiopia, April, 2014.

Variable	*N*	%
Knowing at least three danger signs of pregnancy	190	23.2
Knowing at least one danger sign of pregnancy	533	65.1
Knowing at least three danger signs of labor and delivery	185	22.6
Knowing at least one danger sign of labor and delivery	591	72.2
Knowing at least three danger signs of postnatal period	79	9.6
Knowing at least one postnatal period danger sign	409	49.9

**Table 5:** Proportion of women (*n* = 819) who reported types of key danger signs during pregnancy, childbirth, and postpartum period in South Wollo Zone, Northeast Ethiopia, April, 2014.

Key danger signs	Pregnancy		Labor and delivery		PNC	
	*N*	%	*N*	%	*N*	%
Vaginal bleeding	306	37.4	365	44.6	263	32.1
Severe headache	80	9.8	50	6.1	47	5.7
Blurred vision	49	6	—	—	21	2.5
Convulsion	22	2.7	14	1.7	11	1.3
Swelling of face and feet	86	10.5	—	—	47	5.7
Fever	171	20.9	84	10.2	110	13.4
Loss of consciousness	28	3.4	24	3	—	—
Difficulty of breathing	30	3.7	—	—	14	1.7
Sever weakness	79	9.6	—	—	68	8.3
Severe abdominal pain	148	18	—	—	—	—
Accelerated/reduced fetal movement	166	20.3	—	—	—	—
Water breaks without labor	25	3	—	—	—	—
Prolonged labor	—	—	407	49.7	—	—
Placenta not delivered within 30 minutes	—	—	275	33.6	—	—
Malodorous vaginal discharge					102	12.5

**Table 6:** Proportion of women who know BPCR plan and practice in South Wollo Zone, Northeast Ethiopia, April, 2014.

Variable	*N*	%
Know at least three BPCR plans	218	26.6
Practice at least three BPCR plans	197	24.1
Identify place of delivery	358	43.7
Save money	307	37.5
Prepare essential items for clean and safe delivery	300	36.6
Identify skilled provider	176	21.5
Identify danger signs	66	8
Designate decision maker to her	24	2.9
Identify source of support	28	3.4
Arrange emergency fund	252	30.7
Arrange transport	89	10.8
Arrange blood donor	9	1
Emergency obstetric care (EMOC)	56	6.8

**Table 7:** Proportion of women who heard about BPCR and their source of information in South Wollo Zone, Northwest Ethiopia.

Variable	*N*	%
Participated BPCR related community meeting/pregnant women group discussion (*n* = 819)	498	60.8
Heard about BPCR (*n* = 819)	591	72.2
Source of information (*n* = 591)		
Health care providers	490	82.9
Pregnant women group discussion	172	29.1
Health development army	141	23.9
Mass media	90	15.2

**Table 8:** Factors associated with BPCR practice in South Wollo Zone, Northeast Ethiopia, April, 2014.

Variable	BPCR practice		COR (95% CI)	AOR (95% CI)
	Yes	No		
Education				
Unable to read and write	59	383	**1**	**1**
Primary	57	178	2.07 (1.38, 3.11)	0.86 (0.33, 2.26)
Secondary and above	81	61	8.61 (5.60, 13.26)	6.20 (1.36,28.12)^*∗*^

Lifetime history of stillbirth				
No	126	432	1	1
Yes	25	10	8.57 (4.01, 18.32)	5.80 (1.13,29.63)^*∗*^

Practice ANC for last child pregnancy				
No	47	311	1	1
Yes	104	131	5.25 (3.52, 7.84)	5.44 (2.07,14.27)^*∗*^

Delivery practice for last childbirth				
Home	95	406	1	1
Health institution	104	36	6.64 (4.13, 10.68)	0.37 (0.09, 1.54)

PNC practice for last childbirth				
No	111	420	1	1
Yes	38	22	6.42 (3.65, 11.29)	1.22 (0.31, 4.78)

Trimester				
Second	31	341	1	1
Third	166	281	6.49 (4.29, 9.83)	7.60 (2.82,20.45)^*∗*^

Knowledge of at least three danger signs of pregnancy				
No	93	536	1	1
Yes	104	86	6.69 (4.69, 9.67)	2.13 (0.85, 5.28)

Knowledge of at least three danger signs of labor and delivery				
No	96	538	1	1
Yes	101	84	6.73 (4.69, 9.67)	0.99 (0.39, 2.47)

Knowledge of at least three danger signs of postnatal period				
No	420	22	1	1
Yes	111	38	6.42 (3.65, 11.29)	2.48 (0.65, 9.35)

Women BPCR plan community meeting participation				
No	15	306	1	1
Yes	182	316	11.74 (6.78, 20.35)	4.36 (1.17,16.21)^*∗*^

ANC attendance (visit)				
Less than or equal to one visit	44	505	1	1
Attended 2-3 visits	75	89	9.67 (6.26, 14.94)	6.20 (2.62,14.68)^*∗*^
Attended 4 and above visits	78	28	31.97 (18.81, 15.3)	76.91 (19.02,31.09)^*∗*^

Male partner counseled on BPCR during women ANC follow-up				
No	97	270	1	1
Yes	129	51	7.04 (4.72, 10.49)	4.45 (1.95,10.16)^*∗*^

Remark: ^*∗*^(significant at *P* value < 0.05).

## Abbreviations

AOR: Adjusted odds ratio
HH: Households
OR: Odds ratio
SPSS: Statistical Package for Social Sciences
WHO: World Health Organization.
